# Synbiotic Supplementation Modulates Gut Microbiota, Regulates β-Catenin Expression and Prevents Weight Gain in ob/ob Mice: Preliminary Findings

**DOI:** 10.3390/ijms231810483

**Published:** 2022-09-10

**Authors:** Sebastião Mauro B. Duarte, José Tadeu Stefano, Lucas A. M. Franco, Roberta C. Martins, Bruna D. G. C. Moraes, Denise Frediani Barbeiro, Nathalia Oliveira, Junia Marielle Teixeira Rodrigues Neri, Bruno Cogliati, Denise Siqueira Vanni, Ester C. Sabino, Flair J. Carrilho, Claudia P. Oliveira

**Affiliations:** 1 Laboratório de Gastroenterologia Clínica e Experimental LIM-07, Division of Clinical Gastroenterology and Hepatology, Hospital das Clínicas HCFMUSP, Department of Gastroenterology, Faculdade de Medicina, Universidade de Sao Paulo, São Paulo 05403-000, SP, Brazil; 2 Department of Infectious Diseases, Instituto de Medicina Tropical, Faculdade de Medicina, Universidade de Sao Paulo, São Paulo 05403-000, SP, Brazil; 3 Laboratório de Investigação Médica LIM-51, Faculdade de Medicina, Universidade de Sao Paulo, São Paulo 05403-000, SP, Brazil; 4 Department of Pathology, Escola de Medicina Veterinária e Ciência Animal, Universidade de Sao Paulo, São Paulo 05508-270, SP, Brazil

**Keywords:** gut microbiota, synbiotic supplementation, probiotics, prebiotics, ob/ob mice

## Abstract

Background: Obesity is one of the main health problems in the world today, and dysbiosis seems to be one of the factors involved. The aim of this study was to examine the impact of synbiotic supplementation on obesity and the microbiota in ob/ob mice. Twenty animals were divided into four groups: obese treated (OT), obese control (OC), lean treated (LT) and lean control (LC). All animals received a standard diet for 8 weeks. The treated groups received a synbiotic (Simbioflora-Invictus Farmanutrição Ltd., Sao Paulo, Brazil) in water, while the nontreated groups received only water. After 8 weeks, all animals were sacrificed, and gut tissue and stool samples were collected for mRNA isolation and microbiota analysis, respectively. β-Catenin, occludin, cadherin and zonulin in the gut tissue were analyzed via RT-qPCR. Microbiome DNA was extracted from stool samples and sequenced using an Ion PGM Torrent platform. Results: Synbiotic supplementation reduced body weight gain in the OT group compared with the OC group (*p* = 0.0398) and was associated with an increase in Enterobacteriaceae (*p* = 0.005) and a decrease in Cyanobacteria (*p* = 0.047), *Clostridiaceae* (*p* = 0.026), *Turicibacterales* (*p* = 0.005) and *Coprococcus* (*p* = 0.047). On the other hand, a significant reduction in *Sutterella* (*p* = 0.009) and *Turicibacter* (*p* = 0.005) bacteria was observed in the LT group compared to the LC group. Alpha and beta diversities were different among all treated groups. β-Catenin gene expression was significantly decreased in the gut tissue of the OT group (*p* ≤ 0.0001) compared to the other groups. No changes were observed in occludin, cadherin or zonulin gene expression in the gut tissue. Conclusions: Synbiotic supplementation prevents excessive weight gain, modulates the gut microbiota, and reduces β-catenin expression in ob/ob mice.

## 1. Introduction

Some studies have associated obesity with the gut microbiota (GM), small intestinal bacterial overgrowth (SIBO), intestinal permeability alterations and an increase in lipopolysaccharide (LPS) production [1,2]. These processes induce metabolic endotoxemia, inflammation, impaired glucose metabolism, insulin resistance (IR) and obesity and contribute to the development of metabolic syndrome (MetS) and type 2 diabetes mellitus (T2DM) [3].

The term synbiotic is used to refer to products containing both probiotics and prebiotics, for example, a mixture of fructooligosaccharides (FOS) with lactisol, *Bifidobacteriase* and *Lactobacillus* [4]. These factors promote the survival and implantation of probiotics in the large intestine. Some studies have shown that the consumption of synbiotics prevents bacterial translocation and epithelial invasion and inhibits bacterial adhesion to the mucosa and the production of antimicrobial peptides, thereby reducing inflammation and stimulating host immunity [5,6]. Intestinal colonization of synbiotic bacteria seems to be a good strategy to reduce the damage caused by SIBO and increased gut permeability. Probiotics are living microbial food supplements that promote the maintenance of mucosal integrity [7], the GM balance, increased humoral and cellular immunity and a reduction in cholesterol and triglycerides (TGs) [8,9]. Prebiotics are derived from naturally occurring carbohydrates present in certain vegetables that are not hydrolyzed by digestive enzymes and thus reach the large intestine to be digested by the GM. This type of supplement works as an energy source for the growth of beneficial bacteria [7].

Intestinal cells are connected by multiple junctional complexes, including tight junctions and adherent junctions. Tight junctions are multiprotein complexes found in regions where the membranes of two cells come together, and their main functions are sealing and gating, thereby regulating the passage of molecules and ions between cells [10]. Recently, researchers have demonstrated the participation of β-catenin, an important gene involved in tight junction signaling, in several inflammatory diseases, such as sepsis, colitis, bacterial infection and hepatic and cardiac inflammation, indicating a mediator role of β-catenin in inflammation [11,12,13,14].

Genetically obese mice (ob/ob mice) are characterized by leptin deficiency, which induces hyperphagia, excessive nutrient intake and reduced energy expenditure, leading to the development of MetS, with visceral obesity and IR mimicking human obesity [15]. Due to this phenotype, which facilitates the development of IR, T2DM and the inflammatory response, these animals are commonly used in obesity research [16,17]. Due to the paucity of studies evaluating the supplementation of prebiotics, probiotics and synbiotics in obesity and the lack of standardization in these studies, we carried out a study using ob/ob mice to test the effects of synbiotic supplementation in this experimental model, particularly on the modulation of obesity, GM, gut integrity and intestinal permeability.

## 2. Results

### 2.1. Weighing of Animals

**Figure 1:**
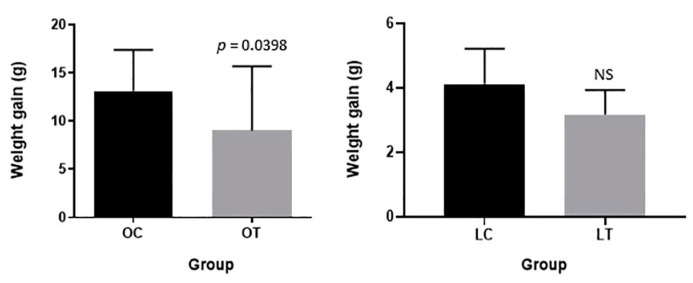
Body weight gain after synbiotic supplementation in obese control (OC), obese treated (OT), lean control (LC) and lean treated (LT) animals. NS: no significance.

Body weight gain after synbiotic supplementation in the obese treated group was significantly lower than that in the obese control group (*p* = 0.0398). No relevant modifications were observed in body weight gain when comparing the lean treated and control groups (Figure 1).

### 2.2. Analysis of Intestinal Microbiota

Microorganisms belonging to the bacterial kingdom were searched and quantified following the taxonomic classification of phyla, class, order, family and genus (species subdivision was not contemplated in the analysis).

**Figure 2:**
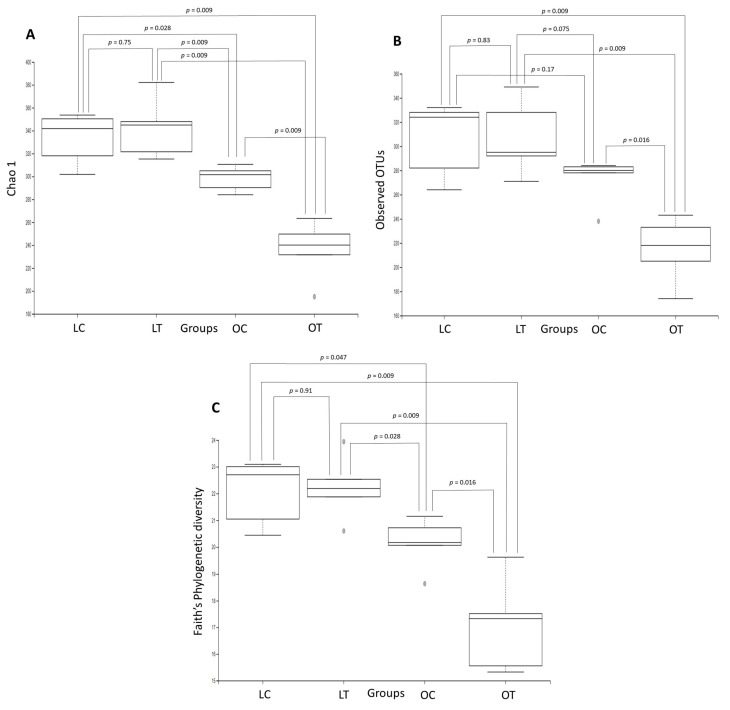
Comparison of the gut microbiota structures in the lean control (LC), lean treated (LT), obese control (OC) and obese treated (OT) groups. Boxplots showing the Chao1 index (**A**), observed OTUs (**B**) and Faith’s phylogenetic tree (**C**) were constructed to evaluate microbiome diversity.

Rarefaction curves showed that the number of observed OTUs plateaued after 54,789 reads, suggesting that we had a good representation of the microbial community. Figure 2 shows boxplots of the microbiome diversity analysis determined by the Chao1 index, observed OTUs, and Faith’s phylogenetic tree. A significant reduction was observed in the alpha diversity indices. Figure 2A compares the Chao1 index between the groups: lean control vs. obese treated (*p* = 0.009), lean control vs. obese control (*p* = 0.028), lean treated vs. obese control (*p* = 0.009), lean treated vs. obese treated (*p* = 0.009) and obese control vs. obese treated (*p* = 0.009). No significant difference was observed between the lean control and the lean treated groups (*p* = 0.75). In the OTU analysis, the following differences were observed: lean control vs. obese treated (*p* = 0.009), lean treated vs. obese treated (*p* = 0.009) and obese control vs. obese treated (*p* = 0.016). No significant difference was observed between the lean treated vs. obese control (*p* = 0.075), lean control vs. lean treated (*p* = 0.83) or lean control vs. obese control (*p* = 0.17) groups (Figure 2B). In Faith’s phylogenetic diversity analysis, differences were observed between the following groups: lean control vs. obese control (*p* = 0.047), lean control vs. obese treated (*p* = 0.009), lean treated vs. obese control (*p* = 0.028), lean treated vs. obese treated (*p* = 0.009) and obese control vs. obese treated (*p* = 0.016). No significant difference was observed between the lean control and the lean treated groups (*p* = 0.91) (Figure 2C). On the other hand, no significant differences were observed in the alpha diversity indices (Shannon, Simpson and Pielou’s evenness) among the groups.

**Figure 3:**
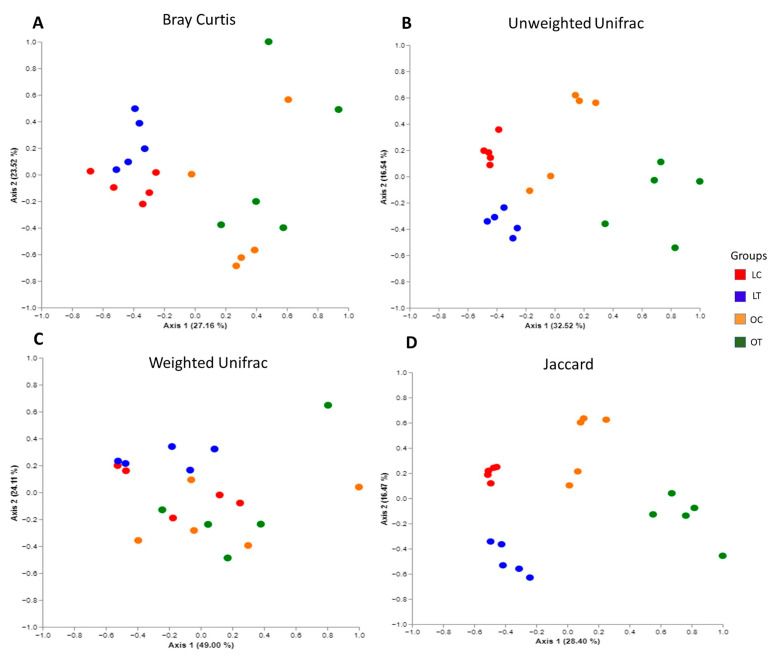
Composition changes in gut microbiota based on PCoA, including Bray Curtis (**A**), unweighted UniFrac (**B**), weighted UniFrac (**C**) and Jaccard (**D**) analyses of the lean control (LC), lean treated (LT), obese control (OC) and obese treated (OT) groups.

The plots revealed that the samples were clustered according to their bacterial composition. PERMANOVA indicated a significant difference in beta diversity between the groups (Bray Curtis: lean control vs. lean treated (*p* = 0.012), lean control vs. obese control (*p* = 0.010), lean control vs. obese treated (*p* = 0.008), lean treated vs. obese control (*p* = 0.009) and lean treated vs. obese treated (*p* = 0.008). No significant difference was observed between the obese control vs. obese treated groups (*p* = 0.111) (Figure 3A). Unweighted UniFrac: lean control vs. lean treated (*p* = 0.012), lean control vs. obese control (*p* = 0.010), lean control vs. obese treated (*p* = 0.008), lean treated vs. obese control (*p* = 0.009) and lean treated vs. obese treated (*p* = 0.008). No significant difference was observed between the obese control vs. obese treated groups (*p* = 0.111) (Figure 3B). Weighted UniFrac: lean treated vs. obese control (*p* = 0.008) and lean treated vs. obese treated (*p* = 0.019). No significant difference was observed between the lean control vs. lean treated (*p* = 0.15), lean control vs. obese control (*p* = 0.209), lean control vs. obese treated (*p* = 0.078) or obese control vs. obese treated (*p* = 0.61) groups (Figure 3C). Jaccard: lean control vs. lean treated (*p* = 0.009), lean control vs. obese control (*p* = 0.011), lean control vs. obese treated (*p* = 0.012), lean treated vs. obese control (*p* = 0.008), lean treated vs. obese treated (*p* = 0.006) and obese control vs. obese treated (*p* = 0.009) (Figure 3D).

**Figure 4:**
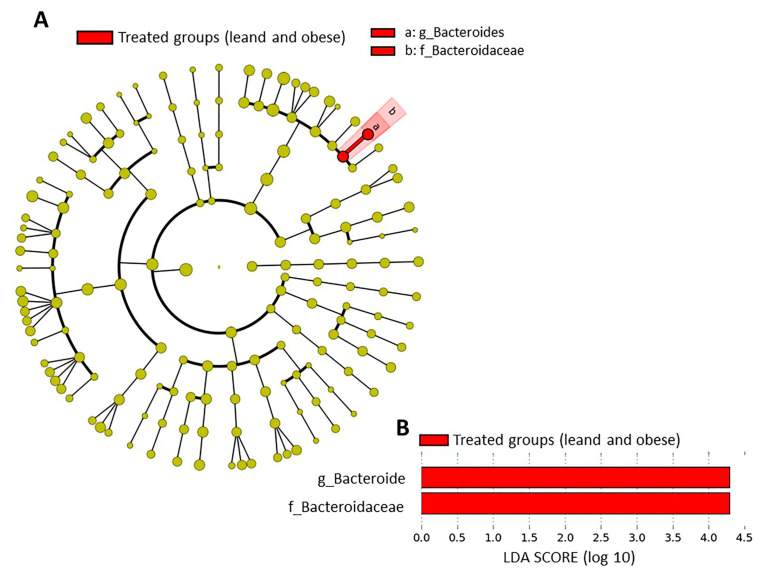
Taxonomic differences in fecal microbiota between groups. Taxa enriched in treated groups (lean and obese) have a positive score (red) (**A**). Only taxa meeting an LDA significance threshold >2 are shown (**B**).

The metagenome analysis LEfSe approach was applied to identify the key phylotypes responsible for the difference between the groups. *Bacteroides* and *Bacteroidaceae*, which were more abundant in the treated groups (lean and obese) than in the controls, were the dominant phylotypes that contributed to the difference between the intestinal microbiota in the treated groups (Figure 4A,B).

**Figure 5:**
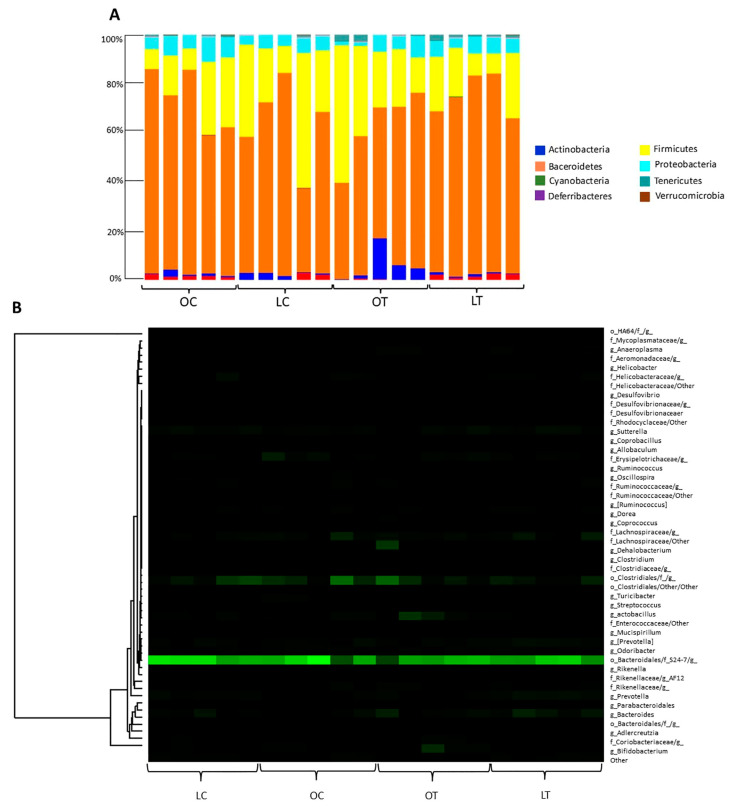
The relative abundance of bacteria under different sample storage conditions is represented by operational taxonomic units (OTUs) according to phyla (**A**). Heatmap of relative abundance at the genus level (**B**). Legend: OC, obese control group; LC, lean control group; OT, obese treated group; LT, lean treated group.

**Figure 6:**
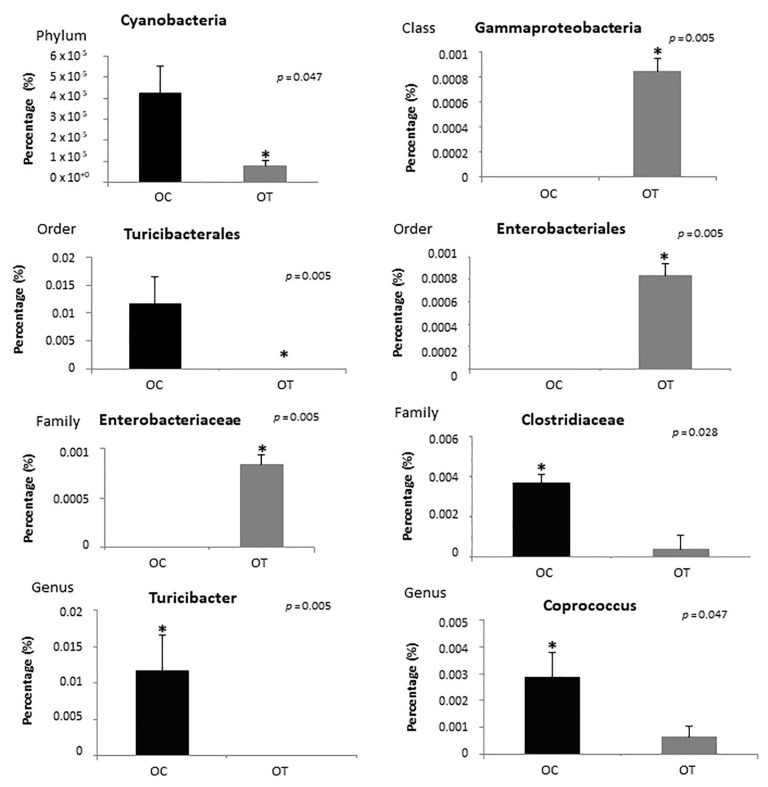
Analysis of the distribution of bacteria in the obese control (OC) and obese treated (OT) groups according to phyla, class, order, family and genus. * Statistical significance.

Regarding the phyla, the proportions of *Bacteroidetes* and *Firmicutes* observed among samples in the obese treated group compared to the obese control group were 71.02% and 18.19%, respectively (Figure 5A). Figure 5B shows a heatmap representation calculated at the genus level of the classified reads obtained. The *Cyanobacteria* phylum and the *Turicibacterales* order were reduced in the obese treated group (*p* = 0.047, *p* = 0.005, respectively), and among bacterial classes there was an increase in *Gammaproteobacteria* (*p* = 0.005), the bacteria order *Enterobacteriales* (*p* = 0.005) and the bacteria family *Enterobacteriaceae* (*p* = 0.005). In addition, the *Clostridiaceae* family (*p* = 0.026) and the bacterial genera *Turicibacter* (*p* = 0.005) and *Coprococcus* (*p* = 0.047) were decreased in the OT group (Figure 6).

**Figure 7:**
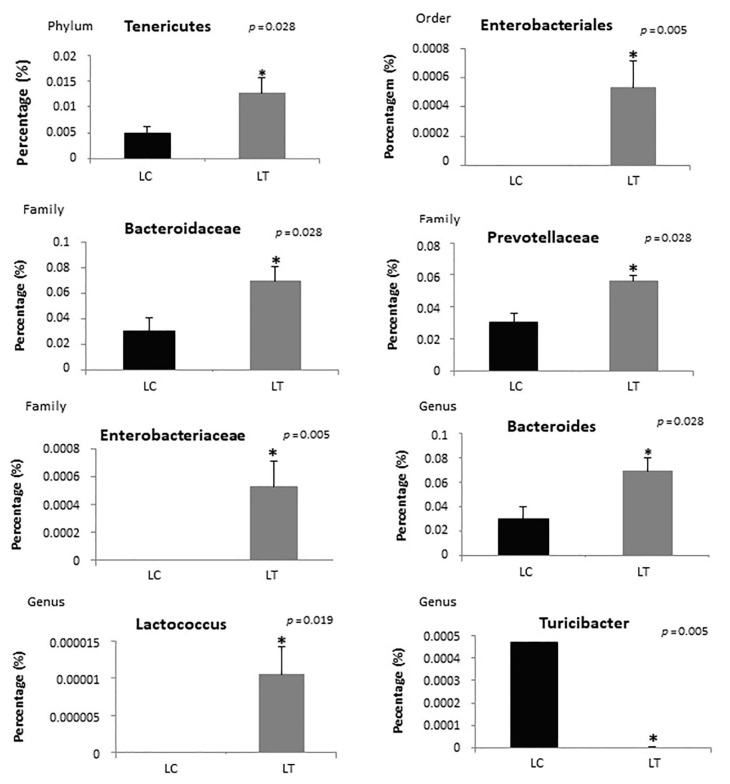
Analysis of the distribution of bacteria in lean control (LC) and lean treated (LT) groups according to phyla, order, family and genus. * Statistical significance.

Analysis of the distribution of bacteria in the lean treated group showed an increase in bacteria in the *Tenericutes* phylum (*p* = 0.028); *Enterobacteriales* order (*p* = 0.005); *Bacteroidaceae* (*p* = 0.028), *Prevotellaceae* (*p* = 0.028), and *Enterobacteriaceae* families (*p* = 0.005); and *Bacteroides* (*p* = 0.028) and *Lactococcus* (*p* = 0.019) genera compared to the control group. There was also a decrease in the *Turicibacter* genus (*p* = 0.005) and *Sutterella* genus (*p* = 0.009) in the treated group (Figure 7).

### 2.3. Gene Expression Analysis

**Figure 8:**
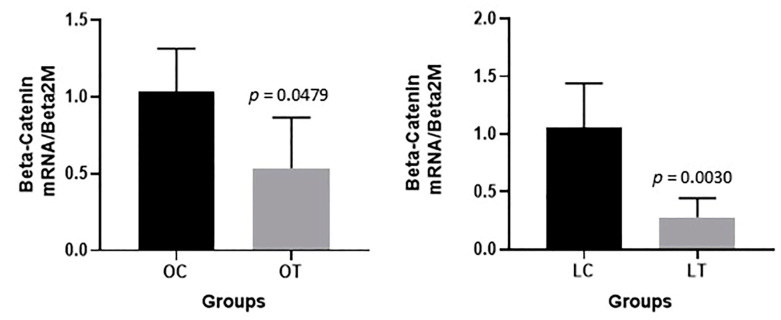
β-Catenin expression in the gut of animals: obese control (OC), obese treated (OT), lean control (LC) and lean treated (LT) groups.

A significant decrease in β-catenin gene expression in the gut tissue was observed in the obese treated group compared to the obese control group (*p* = 0.0479) and in the lean treated group compared to the lean control group (*p* = 0.0030) (Figure 8). On the other hand, no significant changes were verified among the groups in the gene expression levels of cadherin (*p* = 0.4048), occludin (*p* = 0.2063) or zonulin (ZO-1) (*p* = 0.171) in the gut tissue (see Appendix A).

## 3. Discussion

The present study demonstrated that dietary supplementation with synbiotics prevented excessive weight gain in obese mice compared to controls, modulated the GM and reduced β-catenin gene expression. β-Catenin is an important gene involved in tight junction signaling, inflammation and obesity [14].

The mechanism by which synbiotic supplementation influences body weight has not been fully elucidated. However, in keeping with our results, previous studies have demonstrated that dietary supplementation with a combination of probiotics and prebiotics is able to modulate the GM in mice and obese humans, which leads to significant changes in the prevalence of specific intestinal bacteria. With dietary supplementation, these bacterial populations may benefit from the decrease in energy harvest capacity from diet and thus reduce weight gain [18,19].

In the present study, intragroup analyses revealed differing alpha and beta diversities between all treated groups and the control groups. Additionally, the intergroup analyses also showed a significant difference between the lean and obese treated and control groups. Our data demonstrate that the richness and evenness of the GM in lean and obese animals were different before treatment, and this difference became more evident after synbiotic supplementation. In line with our findings, studies of probiotic supplementation in humans and mice have shown changes in GM richness and diversity under conditions such as obesity and metabolic disorders [20,21,22,23]. In addition to synbiotic supplementation, recent studies have shown that traditional Chinese medicine, rich in active ingredients, alkaloids, polysaccharides, glycosides, tannins and enzymes, can significantly adjust GM diversity, stimulating the growth of synbiotic bacteria and inhibiting excessive reproduction of pathogenic bacteria to maintain a healthy intestinal environment [24,25]. Lower diversity in the GM has been linked to obesity, higher IR, higher visceral fat and numerous inflammatory conditions [26]. Thus, GM diversity may be linked to body weight.

The GM is mainly composed of bacteria from the *Bacteroidetes* and *Firmicutes* phyla. *Proteobacteria, Verrucomicrobia, Actinobacteria, Fusobacteria*, and *Cyanobacteria* are present in minor proportions [22]. Adequate amounts of *Cyanobacteria* are considered beneficial for the host to diminish inflammation through NF-κB inhibition and, consequently, reduce proinflammatory cytokine levels, thereby protecting the host against oxidative stress [23]. However, increased *Cyanobacteria* abundance has been associated with obesity [27], and a recent study published by Shao et al. showed that the abundance of these bacteria decreases after weight loss [28]. Our findings showed a reduction in the *Cyanobacteria* phylum in the obese treated group after supplementation, which was associated with weight loss, similar to the findings of Shao et al. [28].

*Turicibacter* is a genus in the *Firmicutes* phylum of bacteria that has been found most commonly in the gut [29]. Although this bacterium has been associated with greater energy extraction from the diet, which might be related to obesity [1], the data in the literature are conflicting. Studies have also shown a negative correlation between the amount of *Turicibacter* and NF-κB and have associated a lower amount of this bacteria with individuals with the highest degree of inflammation, obesity and steatosis [3]. In our study, the analyses of the fecal microbiota of ob/ob mice (obese and lean) after synbiotic supplementation demonstrated a decrease in the *Turicibacter* genus compared to the control groups, which might be responsible for reducing energy extraction from the diet and could be an interesting finding. On the other hand, we observed an increase in the *Enterobacteriaceae* family relative to the control groups. Potential overgrowth of the *Enterobacteriaceae* family is linked to the severity of cirrhosis and its complications, such as hepatic encephalopathy [30].

The other relevant finding of our study was the decrease in the *Clostridiaceae* family and *Coprococcus* genus in the obese treated group and the reduction in the abundance of the *Sutterella* genus in the lean treated group. The *Clostridiaceae* family is a group of bacteria present mainly in obese and T2DM animals [31] and is associated with dysbiosis in adults and children [32] and inflammatory bowel disease in adults [33]. Consistent with our findings, a recent study demonstrated that probiotic supplementation (*Lactobacillus paracasei*) is able to reduce the abundance of *Clostridiaceae* [34]. On the other hand, *Coprococcus*, a genus in the *Firmicutes* phylum, when increased, has been associated with a high-fat diet in mice [35]. Bacteria of the genus *Sutterella* have often been associated with inflammatory bowel disease and disruption of intestinal epithelial homeostasis [36]. It is evident that the consumption of high-protein and high-sugar diets increases *Sutterella* abundance in the gut [37] and that probiotic supplementation reduces *Sutterella* abundance [38], which supports our findings in the lean treated group.

In our study, a significant increase in the following groups of gut bacteria was observed in the LT group: *Bacteroides* and *Lactococcus* genera, *Enterobacteriales* order, *Bacteroidaceae* and *Prevotelaceae* families. The abundance of some of these bacteria is linked to improvement of the integrity of the intestinal barrier [39].

The main role of the intestinal barrier is to separate the internal environment from the luminal content, and the complex system of intercellular junctions, including tight junctions, seals the epithelial cells together to form a continuous layer [10]. In our study, we observed lower expression of intestinal β-catenin in the obese treated and lean treated groups than in the control groups. There were no differences in cadherin, occludin or ZO-1 expression. β-Catenin is one of the proteins that compose tight junctions, which are primarily responsible for maintenance of the intestinal permeability barrier and regulate the passage of ions and solutes between cells via the paracellular pathway [40]. However, when β-catenin expression is increased, it enters the cell nucleus and induces NF-kB activation, leading to the expression of proinflammatory genes and certain oncogenes, which are important in the development of some intestinal diseases [41,42] and hepatocellular carcinoma (HCC) [43].

Studies have shown a relationship between β-catenin signaling pathways and leptin expression. Leptin-activated WNT/β-catenin signaling participates in the neuroendocrine control of glucose homeostasis [44,45]. Our data suggest that the decrease in β-catenin expression observed in both treated groups may be associated with the use of the synbiotic and not only related to the mutation of the leptin gene characteristic of the animal model used. Evidence concerning the effect of synbiotic supplementation on β-catenin modulation is scarce. Our results are consistent with those of Kuugbee et al., who showed inhibition of the β-catenin signaling pathway after probiotic [46] and synbiotic supplementation [14]. Based on these results, we can infer that the reduction in β-catenin expression improves the permeability of the intestinal barrier, preventing the passage of endotoxins from the intestinal lumen through the intestinal barrier and consequently not triggering inflammatory cytokine production, which is important in the development of obesity. On the other hand, studies have shown increased expression of tight junction proteins after synbiotic administration, highlighting ZO-1, occludin and claudin but not evaluating β-catenin [25,47]. Apparently, modulation of intestinal tight junctions occurs with prolonged use of synbiotics, which perhaps justifies the lack of differences in the gene expression levels of cadherin, occludin and ZO-1 observed in our study.

Our study has strengths and limitations that should be considered. The strengths of our study were the combination of four different probiotic strains, the choice of isogenic mice, the microbial sequencing techniques, and the rigorous evaluation of gene expression and liver histology performed by a specialist. However, our study also had some limitations. We chose only one probiotic fiber to include in the synbiotic supplementation, and the treatment period lasted only 8 weeks. Perhaps for these reasons, we did not observe consistent results in gene expression in these animals. Although this model does not exactly reproduce the mechanisms of the disease in humans and there are difficulties in transposing the results observed in experimental studies in animals to humans, it is important to understand the physiological mechanisms involved in the disease because it is isogenic and leads to hyperphagia, reduced energy expenditure, MetS, visceral obesity and obesity. Despite these limitations, we believe that our results are encouraging and support the consideration of larger, well-designed studies to evaluate synbiotic supplementation for obesity prevention.

## 4. Material and Methods

### 4.1. Animals

Twenty adult male ob/ob mice from the vivarium of the Laboratory of Clinical and Experimental Gastroenterology (LIM-07) of the Discipline of Clinical Gastroenterology of the Faculdade de Medicina, Universidade de São Paulo (FMUSP) were housed in a temperature-, humidity-, and ventilation-controlled facility with a 12 h light/dark cycle. All procedures for animal experimentation followed the ethical guidelines of the Helsinki Declaration of 1975 (NIH Publication No. 85-23, revised 1996) and the Guidelines for Animal Experimentation of the FMUSP.

This study was developed at the Laboratório de Gastroenterologia Clínica e Experimental LIM-07, Division of Clinical Gastroenterology and Hepatology, Hospital das Clínicas HCFMUSP, Department of Gastroenterology, FMUSP, Sao Paulo, SP, Brasil in collaboration with the Laboratório de Parasitologia LIM-46 do Instituto de Medicina Tropical da FMUSP, the Laboratório de Emergências Clínicas LIM-51 da FMUSP. During the preparation of the experimental plan, the principles of the 3Rs (replacement, reduction and refinement) governing the ethics of animal use in experiments were addressed [48].

### 4.2. Experimental Procedures

Animals were divided into four experimental groups: obese treated [OT (n = 5)], obese control [OC (n = 5)], lean treated [LT (n = 5)] and lean control (lean mice) [LC (n = 5)]. The OT and LT groups received drinking water with a combination of probiotics and prebiotics [*Lactobacillus acidophilus* SD 5221, 10^9^ colony forming units (CFU); *Lacticaseibacillus rhamnosus* SD 5675 (10^9^ CFU); *Lactobacillus paracasei* SD 5275 (10^9^ CFU); *Bifidobacterium lactis* SD 5674 (10^9^ CFU); and fructooligosaccharides (5.5 g) (Simbioflora-Invictus Farmanutrição Ltd., Sao Paulo, Brazil)] for 8 weeks. The OC and LC groups received only potable water. All groups were given the standard diet Nuvilab CR1 (Nuvital Nutrientes S/A, Colombo, Brazil) (see Appendix B).

Body weight was measured using a digital balance (Gehaka, Model BK4001, São Paulo, Brazil), and weight gain was calculated as the difference between body weight measured at the beginning and at the end of the protocol.

After the treatment period, the animals were anesthetized with ketamine hydrochloride (0.1 mL/kg) administered intraperitoneally and sacrificed. Hepatic tissue samples were collected for histological analysis. The gut tissue samples were collected for analysis of the mRNA levels of genes related to gut integrity [β-catenin (5′ GTGCAATTCCTGAGCTGACA 3′-5′ CTTAAAGATGGCCAGCAAGC 3′), occludin (5′ CCTCCAATGGCAAAGTGAAT 3′-5′ CTCCCCACCTGTCGTGTAGT 3′), cadherin (5′ ACTGTGAAGGGACGGTCAAC 3′-5′ TGTCCCGGGTATCATCATCT 3′) and zonulin (5′ CCACCTCTGTCCAGCTCTTC 3′-5′ CACCGGAGTGATGGTTTTCT 3′)]. Gene expression was evaluated via real-time quantitative reverse transcription polymerase chain reaction (RT-qPCR). The beta-2 microglobulin (β2M) (5′ CCAGCGUACUCCAAAGAUUTT 3′-5′ AAUCUUUGGAGUACGCUGGTT 3′) gene was used as an internal control.

### 4.3. Gene Expression

#### 4.3.1. Extraction of RNA from Gut Tissue

For extraction, the gut tissue was fragmented into a tissue sprayer (Micro-Dismenbrenator II B. Braun Biotech International, Melsungen, Germany). To the pulverized material, 1 mL TRIzol^®^ (Invitrogen Life Technologies, Carlsbad, CA, USA) was added, and the solution was incubated for 5 min at room temperature. To the solution, 200 μL of chloroform (Merck, Darmstadt, Germany) was added, and the solution was vigorously stirred for 15 s, followed by incubation for 5 min at room temperature and centrifugation for 15 min at 12,000 rpm at 4 °C (Eppendorf 5417-R, Hamburg, Germany). The supernatant was then transferred to a new 1.5 mL sterile tube, and RNA was precipitated with 500 μL of isopropanol (Merck, Darmstadt, Germany).

The mixture was allowed to stand for 10 min and then centrifuged for 15 min at 12,000 rpm at 4 °C (Eppendorf 5417-R, Hamburg, Germany). The supernatant was removed, and the RNA pellet was washed with 1 mL of 70% ethanol (Merck, Darmstadt, Germany), centrifuged again and resuspended in 200 µL of sterile water [(UltraPure™ DNase/RNase-Free Distilled Water (Invitrogen Life Technologies, Carlsbad, CA, USA)].

#### 4.3.2. Quantification and Analysis of Total RNA Integrity

The concentration of total RNA extracted was determined via spectrophotometry [NanoDrop ND-1000 (NanoDrop Technologies, Wilmington, DE, USA)]. The RNA preparation was considered protein free when the A260/280 ratio was between 1.8 and 2.0. For samples that did not reach these values, purifications were performed using a RNeasy™ Mini Kit (Qiagen, Hilden, Germany). The integrity and purity of the RNA were analyzed via 1% agarose gel electrophoresis. Only samples whose bands corresponded to ribosomal RNA (rRNA) 18 and 28S were shown to be intact for analysis under ultraviolet light. The RNA samples were maintained at −80 °C until use.

#### 4.3.3. Real-Time PCR

One hundred nanograms were used for real-time PCR analysis. PCR was performed in a 15 μL reaction mixture containing 7.5 μL 2 × SYBR Green Reaction Mix (Invitrogen Life Technologies, Carlsbad, CA, USA), 0.3 μL each primer (10 pmol), 0.3 μL Super Script III RT/Platinum Taq Mix (10 pmol/μL), 0.15 μL ROX Reference Dye and 5 μL sample in water. Gene-specific primers were used. Reactions were performed using a StepOne™ Real-Time PCR System (Applied Biosystem, Foster City, CA, USA).

#### 4.3.4. Analysis of Fecal Microbiota

##### Fecal Sample Collection, DNA Extraction and Sequencing

The stool samples (n = 20) collected were mixed with RNAlater (Life Technologies Corporation, Carlsbad, CA, USA) and stored at −20 °C. After a maximum period of four hours, they were separated into aliquots of 200 mg each and stored at −80 °C, while sequencing was initiated over a period of approximately twenty-four hours. We used 0.25 g of each stool sample for analysis.

Approximately 0.25 g of feces was used for DNA isolation using a DNeasy PowerSoil Kit (Qiagen, Germantown, MD, USA) following the manufacturer’s instructions. The extracted DNA was quantified with a Qubit^®^ 4.0 fluorometer using a dsDNA HS Assay kit (Invitrogen™, Waltham, MA, USA) according to the manual. PCR amplification was then carried out using primers for the V4 region of the 16S rRNA gene [bacterial/archaeal primer set-515F (5′-CACGGTCGKCGGCGCCATT-3′)/806R (5′-GGACTACHVGGGTWTCTAAT-3 [49]]. The amplification was confirmed by electrophoresis in a 1.5% agarose gel with 1X TAE buffer. Template preparation was performed with an Ion Chef System (Thermo Fisher Scientific, Waltham, MA, USA) using an Ion PGM Hi-Q View Chef Kit. Sequencing was performed in an Ion Personal Genome Machine (PGM) using an Ion PGM Hi-Q Sequencing Kit and Ion 318 Chip v2 following the instructions of the manufacturer (Thermo Fisher Scientific, Waltham, MA, USA). Samples beneath 85,000 reads were resequenced.

##### Analysis of Results

Data analyses were conducted using the Quantitative Insights Into Microbial Ecology (QIIME) software package v1.8 [50]. Reads were filtered by length (>200 bp), quality (Phred Score = 30) and minimum expected error (0.1) utilizing the USEARCH tool [51]. The remaining sequences were grouped into operational taxonomic units (OTUs) based on 97% similarity using the UCLUST algorithm. Singletons were removed. OTUs were classified taxonomically using the Greengenes 16S reference database v. 13.8 [52]. Alpha-diversity indices (Shannon, Simpson, Chao1, observed OTUs, Faith’s phylogenetic diversity and Pielou’s evenness) and beta diversity indices (weighted UniFrac, unweighted UniFrac, Bray Curtis and Jaccard) were calculated based on the rarefied OTU table using 54,789 sequences per sample. The principal coordinates analysis (PCoA) plot for each of the beta diversity indices was generated using Emperor [53]. Compositions of microbiota communities were summarized by proportion at different taxonomy levels, including genus, family, order, class and phylum ranks. A Kruskal-Wallis test was performed to explore differences in the alpha diversity index. Differences in community composition (beta diversity) were assessed using permutational multivariate analysis of variance (PERMANOVA). Kruskal-Wallis and PERMANOVA analyses were corrected using the Benjamini and Hochberg method.

Microorganism features distinguishing fecal microbiota were identified using the linear discriminant analysis (LDA) effect size (LEfSe) method for biomarker discovery, which emphasizes both statistical significance and biological relevance (metagenomic biomarker discovery and explanation). LEfSe applies a Kruskal–Wallis rank-sum test with a normalized relative abundance matrix to detect features with significantly different abundances between assigned taxa and uses LDA to estimate the effect size of each feature.

##### Statistical Analysis

All data are expressed as the mean or median (depending on the distribution pattern of the variables). Minimum (Min), maximum (Max) and standard deviation (DP) values were set. For Gaussian distribution variables, we used a t test, one-way ANOVA and a Newman-Keuls posttest for multiple comparisons. For non-Gaussian distribution variables, Mann-Whitney, Kruskal-Wallis and Dunn’s multiple-comparison tests were used. Chi-square tests and Fisher’s exact test were used to compare histological scores between the groups. A *p* value < 0.05 was considered significant. The SPSS 17.0 software (SPSS Inc., Chicago, IL, USA) was used to perform all calculations and prepare the graphs.

## 5. Conclusions

In conclusion, our experimental study with an animal model shows that synbiotic supplementation is effective in preventing excessive weight gain, positively modulating the GM, and reducing β-catenin expression but was not able to improve the expression of other tight junction genes. Our data provide evidence of the beneficial effects of synbiotic supplementation on obesity prevention. Nonetheless, in the future, more randomized controlled trials of synbiotic supplementation should be performed to explore the mechanisms of the GM and the role of microbe metabolites in obesity.

## Figures and Tables

**Figure A1:**
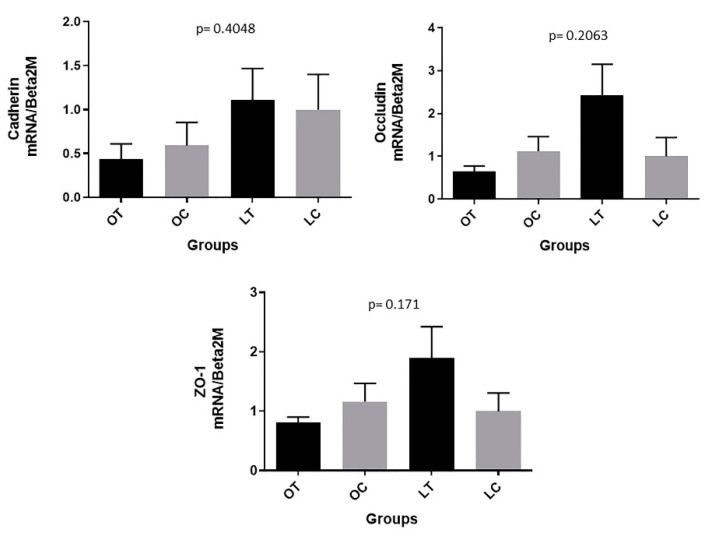
Cadherin, occludin and zonulin (ZO-1) expression in the gut of animals: obese control (OC), obese treated (OT), lean control (LC) and lean treated (LT) groups.

## Appendix B

**Table A1:** Nutritional composition of the Nuvilab CR-1 Autoclavable animal feed (Nuvital Nutrientes S/A, Colombo, Brazil).

Nutrients	Concentration/KG of Product
Protein (min.)	220 g
Ethereal Extract (min.)	40 g
Mineral Material (max.)	90 g
Fibrous Matter (max.)	70 g
Calcium (mín.–max.)	10–14 g
Phosphor (min.)	8000 mg
Vitamin A	25,200.00 UI
Vitamin D3	2100.00 UI
Vitamin E	60.00 mg
Vitamin K3	12.50 mg
Vitamin B1	14.40 mg
Vitamin B2	11.00 mg
Vitamin B6	12.00 mg
Vitamin B12	60.00 mcg
Niacin	60.00 mg
Pantothenic acid	112.00 mg
Folic acid	6.00 mg
Biotin	0.26 mg
Coline	1100.00 mg
Iron	50.00 mg
Zinc	60.00 mg
Copper	10.00 mg
Iodine	2.00 mg
Manganese	60.00 mg
Selenium	0.05 mg
Cobalt	1.50 mg
Lysine	100.00 mg
Methionine	300.00 mg
